# Insurance policy and cropping structure adjustment toward staple grains in China: implications for food system resilience and security

**DOI:** 10.3389/fnut.2025.1636296

**Published:** 2025-08-20

**Authors:** Jiawei Wang, Zihui Yuan, Zhihua Wu, Xia Kuang, Feng Ye

**Affiliations:** ^1^School of Economics, Management and Law, Jiangxi Normal University of Science and Technology, Nanchang, China; ^2^Digital Faculty of Economics, Jiangxi Open University, Nanchang, China; ^3^School of Economics and Management, Jiangxi Agricultural University, Nanchang, China

**Keywords:** agricultural insurance, cropping structure adjustment, staple crop security, digital technology training, distance to administrative center

## Abstract

**Introduction:**

The ongoing decline in staple crop acreage and the accelerating trend of “non-grain” cultivation pose structural risks to China’s food security. Agricultural insurance, beyond its traditional role in risk mitigation, may serve as a structural policy tool to influence farmers’ planting decisions and guide cropping structures toward staple grains. However, empirical evidence on this guiding function remains limited.

**Methods:**

Drawing on data from the 2020 China Rural Revitalization Survey (CRRS), this study employs a two-limit Tobit model to examine the impact pathways and moderating mechanisms through which agricultural insurance affects farmers’ cropping structure adjustment. The analysis addresses endogeneity issues and explores heterogeneous effects across different farmer groups.

**Results:**

Agricultural insurance significantly increases the proportion of staple crops in total cultivated area, with findings robust to both robustness checks and endogeneity tests. Mechanism analysis shows that digital technology training significantly strengthens the incentive effect by improving farmers’ ability to access and interpret policy information. The marginal effect also rises with greater distance from the administrative center, indicating higher responsiveness in institutionally underserved areas. Heterogeneity analysis reveals stronger effects among highly educated farmers, small-scale operators, residents in pilot regions, those in non-plain terrains, and farmers in eastern provinces.

**Discussion:**

These results confirm the effectiveness of agricultural insurance as a policy instrument for guiding cropping structure adjustments. Strengthening policy communication, expanding service coverage—especially in remote areas—and improving coverage levels can further enhance its contribution to national food security strategies and the Sustainable Development Goals (SDGs).

## 1 Introduction

China’s ongoing shift in agricultural land use—marked by a significant and concerning decline in the share of staple crops within total cultivated area, coupled with the expansion of non-grain cultivation—is primarily driven by profit-oriented crop choices (1). This structural transformation poses fundamental threats to the sustainability of food production and the long-term stability of the national food supply. Crucially, staple grains not only serve as the caloric foundation of food security but are also vital for ensuring dietary diversity and fulfilling essential micronutrient requirements, particularly for vulnerable populations (2). Accordingly, the reduction in staple grain cultivation poses direct risks to nutritional security and diet quality. Ensuring adequate staple grain production is therefore critical to achieving Sustainable Development Goal (SDG) 2 (Zero Hunger—specifically targets 2.1 on universal food access and 2.2 on ending all forms of malnutrition) and SDG 12 (Responsible Consumption and Production—specifically target 12.2 on the sustainable management and efficient use of natural resources).

Beyond macro level food security, insurance-induced cropping adjustments directly affect household dietary diversity and food affordability, which are central to SDG 2 and SDG 12. In developing countries such as China, cereals including rice, wheat, and maize account for more than 50 to 60% of rural caloric intake. They provide the primary source of household energy supply but contribute only limited amounts of essential micronutrients such as vitamin A, iron, and zinc (2, 3) When agricultural insurance encourages farmers to maintain or expand staple grain cultivation, it stabilizes household caloric availability and shields rural families from market-driven price volatility, which improves the affordability of basic diets. At the same time, more secure and larger scale grain production increases household income (4). The additional resources can then be used to cultivate cash crops or to purchase nutrient rich foods, which improves dietary diversity. Through this dual pathway, agricultural insurance serves as both a risk mitigation instrument and a nutrition sensitive policy tool that promotes sustainable and resilient food systems.

A critical unresolved issue at the intersection of agricultural policy and economics is how to employ institutional tools that can effectively incentivize farmers to maintain or increase staple crop production in the face of stronger economic returns from non-grain alternatives (5). In this context, agricultural insurance has emerged as a promising structural policy tool with the potential to influence farmers’ cropping decisions beyond its traditional role in risk mitigation (5, 6). By reshaping the perceived risk–return profile of staple grain cultivation, agricultural insurance may help guide farmers toward more food-security and nutrition-sensitive production choices.

However, significant empirical and theoretical gaps remain concerning the role of agricultural insurance in cropping structure adjustments (7). In particular, existing research provides limited insight into how agricultural insurance, through the dual pathways of risk mitigation and policy incentives, can guide farmers to increase their cultivation of staple crops. Whether and how agricultural insurance can effectively mitigate production uncertainty and promote a grain-oriented cropping structure remains a core, unresolved academic and policy question that this study aims to address. Answering this question is essential not only for advancing theoretical understanding of agricultural insurance as a structural policy instrument but also for designing effective interventions to stabilize staple food production—a cornerstone of nutritional security and sustainable agri-food systems aligned with SDGs 2 and 12.

To address these gaps, this study draws on data from the 2020 China Rural Revitalization Survey (CRRS) to examine the impact of agricultural insurance on farmers’ cropping structure adjustments. It focuses on the pathways linking risk mitigation and production structure and further explores moderating mechanisms and outcome heterogeneity. The findings aim to inform the design of more effective insurance-based interventions that support staple grain cultivation, enhance nutritional security, and promote sustainable land use aligned with the targets of SDGs 2 and 12. The structure of the study is organized as follows: Section 2 reviews relevant literature, constructs the theoretical framework, and proposes research hypotheses. Section 3 describes the data source, variable definitions, and model specifications. Section 4 presents the empirical results, including robustness checks, endogeneity tests, moderation analysis, and heterogeneity analysis. Section 5 concludes the study and provides policy recommendations.

## 2 Literature review and theoretical framework

### 2.1 Literature review

The adjustment mechanism of farmers’ planting structure is a core issue in agricultural economics research. For a long time, the academic community has constructed a multi-dimensional explanatory framework around its influencing factors, which can be mainly summarized into three categories: policy and institution, market mechanism, and resource endowment. First, focusing on the external constraints of policies and institutions, it emphasizes that government intervention directly affects crop selection by reshaping farmers’ cost–benefit structure. For example, the price support policy reduces the market risks faced by farmers through the “separation of price and subsidy” mechanism (8), and clear and stable land tenure is considered an important institutional basis for stimulating grain production (9). However, some studies also point out that certain industrial policies oriented toward economic incentives (such as “one village, one product”) may induce farmers to irrationally allocate land resources, leading to the distortion of the crop structure (10). Second, it emphasizes the flexible adjustment role of market factors in crop selection. On the one hand, land transfer and large-scale operation help to spread the costs of mechanical operations and management, thereby increasing the marginal revenue of staple food crops and promoting the “grain-oriented” trend of the planting structure (11, 12). On the other hand, with the outflow of rural labor and the intensification of farmers’ part-time employment, the agricultural labor force is becoming increasingly scarce, prompting farmers to allocate resources to crops with lower labor intensity or higher economic returns, showing a “non-grain” trend (13). Third, from the perspective of resource endowment, it points out the fundamental constraint of natural conditions on the adjustment of the planting structure. For example, fragmented farmland raises the cost of mechanization and inhibits the efficiency of grain production (14); through farmland consolidation and improvement of suitability for mechanization, it is expected to improve this structural disadvantage (15). In addition, environmental factors such as irrigation conditions and climate change also affect farmers’ perception of planting risks and crop allocation behavior (16, 17).

Based on the above research, in recent years, the potential role of agricultural insurance, as an important institutional incentive tool, in the adjustment of the crop structure has begun to attract attention. Initially, the core function of agricultural insurance was post-disaster compensation, and its main goal was to alleviate the natural disasters and market uncertainties faced by farmers (18). However, with the continuous optimization of the institutional design and the continuous strengthening of financial support, its policy function is shifting from a single security mechanism to a behavior-guiding mechanism (19). On the one hand, agricultural insurance reduces the production risks of staple food crops and improves the stability of farmers’ expected income from staple food planting, thereby enhancing its attractiveness (7). Especially under China’s policy-oriented agricultural insurance system, staple food crops such as rice, wheat, and corn generally enjoy a higher subsidy ratio and clearer claim-settlement rules. This differential protection is interpreted by farmers as a “policy encouragement signal” in practice, strengthening the logic of their crop allocation toward staple foods (20).

Meanwhile, existing studies also point out that the incentive effect of agricultural insurance often varies significantly due to differences in farmers’ individual characteristics and environmental conditions (21). Among them, digital technology training, as an effective means to improve farmers’ ability to receive and understand policy information (22), is considered to help enhance their perception and willingness to respond to the agricultural insurance system. Relevant studies have found that farmers with digital skills often have stronger information-acquisition and system-recognition abilities when facing agricultural policy tools and thus are more likely to accept the insurance system and show more positive response behavior in decision-making (23). In addition, as a spatial institution, the institutional effect of agricultural insurance is also limited by the accessibility of policy resources in the geographical location where farmers are located. The distance from the administrative center is an important indicator to measure the accessibility of institutional services. Existing studies have pointed out that agricultural insurance has an obvious spatial spill-over effect (24), although some studies believe that farmers in remote areas may weaken their response ability to agricultural insurance due to information asymmetry, poor service accessibility, and a weak trust mechanism (6, 25). However, some studies also emphasize that precisely because of the scarcity of institutional resources and the lack of alternative mechanisms, farmers in remote areas show higher institutional dependence and response sensitivity, and the marginal incentive effect of agricultural insurance may be more significant in such areas (26).

Overall, agricultural insurance is gradually evolving from a “risk-transfer tool” to a “structural-regulation tool,” and its institutional role in farmers’ crop allocation is becoming an important direction for policy research and empirical evaluation. However, existing studies still face two deficiencies: First, there is a lack of in-depth analysis of systematically incorporating agricultural insurance into the logic of farmers’ planting structure adjustment; second, there is still a lack of systematic discussion on the differences in the role of institutional tools under different farmer capabilities and policy accessibility. To make up for the deficiencies in mechanism integration and action logic in the above research, it is necessary to further sort out the path of agricultural insurance affecting the planting structure at the theoretical level and put forward testable research hypotheses.

### 2.2 Theoretical framework

#### 2.2.1 The impact of agricultural insurance on farmers’ cropping structure adjustment

Against the backdrop of widespread exposure to natural disasters and market volatility, farmers tend to exhibit risk-averse behavior in their crop selection, favoring relatively stable crops to minimize potential losses (6, 27). Agricultural insurance, as an institutionalized risk management tool, provides compensation for yield or income losses, effectively reducing farmers’ concerns about production risks and improving their expectations regarding income stability. This, in turn, enhances their willingness to cultivate staple crops (7). Within China’s policy-based agricultural insurance system, staple crops as rice, wheat, and maize not only receive higher premium subsidies from the government but also enjoy progressively improved coverage. Initially, insurance covered only basic material costs, but has since expanded to include land and labor costs—offering full-cost coverage—and even covers income losses caused by price fluctuations (28). These high-coverage, high-subsidy arrangements affect farmers’ cropping choices in two key ways: First, by mitigating natural and market risks, they reduce the income volatility of staple crops, allowing farmers to make bolder and long-term investment decisions based on more stable return expectations. Second, the structure of the insurance subsidies itself conveys a clear policy signal. By offering differentiated levels of protection for different crops, the government explicitly communicates its institutional preference for staple grain production (20). In recent years, as economic crops such as soybeans and oilseeds have experienced increased income volatility and greater market risk, the comparative advantage of staple crops in terms of insurance protection has become more pronounced. As a result, farmers have become more inclined to avoid risk, follow policy signals, and allocate land resources preferentially to staple crop production (28).

It is thus evident that agricultural insurance influences farmers’ cropping structure through both stabilizing return expectations and signaling policy preferences, guiding farmers to adjust their cropping structures and reinforcing the production priority of staple crops. Based on this theoretical reasoning, we propose the following hypothesis:

*H1*: Agricultural insurance promotes a grain-oriented adjustment in farmers’ cropping structure.

#### 2.2.2 The moderating effect of digital technology training

The extent to which agricultural insurance can effectively influence farmers’ cropping decisions largely depends on their ability to access and understand relevant policy information. In practice, the transmission of institutional incentives is shaped not only by the level of insurance coverage and subsidies but also by whether farmers possess the digital skills needed to receive, interpret, and act upon such information (22, 23). Digital technology training—particularly in basic computer and mobile Internet skills—provides farmers with a practical pathway to enhance their policy awareness and responsiveness. On the one hand, farmers who have received such training are more likely to access official policy notices, enrollment guidelines, and benefit details via mobile phones or the Internet, thereby improving the timeliness and efficiency of information acquisition (22). In this process, policy incentives shift from being “passively received” to “actively sought,” making farmers more likely to view agricultural insurance as a credible institutional arrangement and to adjust their production behavior accordingly. On the other hand, when identifying the scope of coverage and target beneficiaries, digitally trained farmers tend to understand the details more quickly and make more accurate judgments. As a result, they are more likely to align their crop choices with policy priorities, favoring staple crops with higher protection levels (22).

Although digital training does not directly alter production structure, it enhances farmers’ responsiveness to agricultural insurance by improving their access to and interpretation of policy information, thereby amplifying the effect of institutional incentives on cropping behavior. Based on this logic, we propose the following hypothesis:

*H2*: Digital technology training positively moderates the impact of agricultural insurance on grain-oriented cropping structure adjustment.

#### 2.2.3 The moderating effect of distance to the administrative center

Distance to the county administrative center is widely regarded as a key indicator of institutional accessibility and service reach. It has traditionally been seen as a negative factor, reflecting the spatial frictions in policy transmission. However, recent studies suggest that in areas where institutional resources are scarce and alternative support mechanisms are lacking, farmers in remote regions may exhibit stronger dependence on policy tools and higher responsiveness (6). On the one hand, longer distances from administrative hubs are often associated with higher environmental risks and weaker agricultural infrastructure (29), thereby increasing uncertainty in agricultural production. Under such conditions, agricultural insurance becomes a primary tool for mitigating high-risk exposure (21). Farmers are more likely to adjust their crop allocation in response to the stability offered by institutional protection.

On the other hand, due to underdeveloped markets and limited commercial insurance options, policy-based agricultural insurance often assumes a stronger public welfare role in remote or marginalized areas (30). In the absence of viable private alternatives, such institutional tools take on quasi-public goods characteristics, enhancing their incentive effects and prompting farmers to respond more actively to policy signals by optimizing their cropping structure.

Therefore, although administrative distance represents a spatial barrier to policy transmission on the surface, it may actually enhance farmers’ behavioral responses due to heightened policy salience. Based on this logic, we propose the following hypothesis:

*H3*: Distance to the county administrative center positively moderates the effect of agricultural insurance on grain-oriented cropping structure adjustment.

## 3 Research design

### 3.1 Data source

This research relies on data derived from the 2020 wave of the China Rural Revitalization Survey (CRRS), a large-scale household investigation with national representativeness, conducted by the Chinese Academy of Social Sciences. The survey covers multiple dimensions of rural transformation, such as demographics, workforce composition, industrial patterns, household earnings, social protection, consumption behavior, local governance, and institutional change. Conducted biennially, the inaugural round was carried out between August and September 2020 across 10 provincial-level regions, namely, Guangdong, Zhejiang, Shandong, Anhui, Henan, Heilongjiang, Guizhou, Sichuan, Shaanxi, and Ningxia. The dataset comprises inputs from 50 county-level jurisdictions and 156 townships, with 300 village-level and over 3,800 household-level questionnaires collected, totaling information on approximately 15,000 individuals. To ensure geographic diversity and sample validity, the research team applied stratified random sampling strategies that accounted for regional economic disparities, geographic variation, and agricultural features. The selected provinces spanned four major regions—eastern, central, western, and northeastern China. Subsequent county selection was performed through systematic sampling based on local per capita GDP, while township and village selection further reflected development status. Final household respondents were randomly chosen from village rosters to ensure representativeness. After eliminating entries with missing or anomalous values, the refined dataset includes 2,367 valid household responses.

### 3.2 Variable description

1. Dependent variable: Cropping structure adjustment. This study uses the proportion of land allocated to the three major staple crops—wheat, rice, and maize—out of the total sown area to measure farmers’ cropping structure. This indicator reflects farmers’ orientation and structural adjustments between staple and non-staple crops.
2. Key explanatory variable: Agricultural insurance. This binary variable equals 1 if the farmer purchased agricultural insurance for at least one of the three staple crops (wheat, rice, or maize) and 0 if no such coverage was purchased.
3. Moderating variables: Digital technology training and distance to county government. Digital training is measured by whether the respondent has received basic training in computer or mobile Internet use. A value of 1 indicates training was received; otherwise, the value is 0. This variable reflects the farmer’s information access and technological receptivity, enabling analysis of policy responsiveness under different informational conditions. Distance to county government is measured by the geographical distance (in kilometers) between the village committee and the county administrative center. This variable captures spatial differences in public service accessibility and policy delivery efficiency.
4. Instrumental variable: Natural disaster experience. Whether the village experienced natural disasters in the past 3 years is used as an instrument. On the one hand, the frequency of disasters significantly influences both policy design and farmers’ insurance enrollment intentions, satisfying the relevance condition. On the other hand, disaster occurrence is assumed to be unrelated to farmers’ cropping structure decisions, satisfying the condition of exogeneity for a valid instrumental variable.
5. Control variables: Following existing literature, we include variables at the household head, household, and village levels to reduce estimation bias. Household head characteristics include gender, age, education level, and whether the individual holds a village-level position. Household-level characteristics include household size, total cultivated area, and whether the household is classified as low-income. Village-level controls include parcel delivery accessibility, suburban location status, and total collective village assets. Descriptive statistics are shown in Table 1.

**Table 1:** Summary statistics of variables.

Variable category	Variable name	Definition and coding	Mean	Std. dev.
Dependent variable	Cropping structure	Proportion of land sown with wheat, rice, and maize	0.670	0.376
Key explanatory variable	Agricultural insurance	Whether insured for at least one staple crop (No = 0; Yes = 1)	0.395	0.489
Moderating variable	Digital technology training	Trained in computer/mobile Internet use (No = 0; Yes = 1)	0.076	0.264
	Distance to county government	Distance from village committee to county government (km)	25.105	17.280
Instrumental variable	Natural disaster experience	Experienced natural disaster in past 3 years (No = 0; Yes = 1)	0.575	0.494
Control variable	Gender	Female = 0; Male = 1	0.952	0.213
	Age	Age of household head (in years)	55.038	10.575
	Education level	1 = No schooling; 2 = Primary; 3 = Junior High; 4 = High School/Vocational; 5 = College or above	2.66	0.843
	Village official	Holds village-level position (No = 0; Yes = 1)	0.185	0.389
	Household size	Number of household members	4.159	1.536
	Total cultivated area	Household-managed farmland (in hundreds of mu)	0.295	0.889
	Low-income household	Whether household is classified as Dibao (No = 0; Yes = 1)	0.103	0.304
	Parcel delivery accessibility	1 = All households accessible; 2 = Partial; 3 = Not accessible	2.233	0.831
	Urban suburb status	Whether village is located in a suburban area (No = 0; Yes = 1)	0.170	0.376
	Village collective assets	Total assets of the village collective (in 10 million RMB)	0.494	0.707

### 3.3 Model specification

This study investigates the effect of agricultural insurance on farmers’ cropping structure adjustment. The dependent variable—cropping structure—is defined as the proportion of land sown with the three major staple crops (wheat, rice, and maize) relative to the farmer’s total sown area. This is a bounded proportion variable with values ranging from 0 to 1. Given that a substantial number of observations lie at the boundary values (0 or 1), the variable exhibits clear upper and lower censoring.

Using ordinary least squares (OLS) regression for such a variable could yield predictions outside the admissible range and ignore censoring behavior, leading to biased estimates. Although the fractional logit model is suitable for proportion data, it may underestimate boundary effects when a substantial portion of observations lie at the extremes. Therefore, this study employs a two-limit Tobit model to more accurately estimate the effect of agricultural insurance.

The baseline specification is as follows. Let $y_{i}^{*}$ denotes the latent (ideal) level of cropping structure adjustment for farmer iii, modeled as


(1) $$y_{i}^{*}=\alpha_{0}+\beta_{1}{\text{insu}}_{i}+\beta_{2}X_{i}+\varepsilon_{i},\varepsilon_{i}\sim N(0,\sigma^{2})$$

The observed variable $y_{i}\in [0,1]$ is defined based on censoring as


(2) $$y_{i}=\{\begin{array}{c} 0,\text{if}\;y_{i}^{*}\leq 0\\ y_{i}^{*},\text{if}\;0<y_{i}^{*}<1\\ 1,\text{if}\;y_{i}^{*}\geq 1 \end{array}$$

where ${\text{insu}}_{i}$ is a binary variable indicating whether the farmer is covered by agricultural insurance, and $X_{i}$ is a vector of control variables, including individual, household, and village characteristics. $\alpha_{0}$ is the constant term; $\beta_{1}$ and $\beta_{2}$ are the parameters to be estimated. The error term $\varepsilon_{i}$ is assumed to follow a normal distribution.

To explore how the effects of agricultural insurance may vary under different conditions, this study introduces interaction terms between the key explanatory variable and two moderating variables. The extended model is specified as


(3) $$y_{i}^{*}=\alpha_{0}+\beta_{1}{\text{insu}}_{i}+\beta_{2}X_{i}+\beta_{3}({\text{insu}}_{i}*Z_{\mathrm{ni}})+\beta_{4}Z_{i}+\varepsilon_{i}$$

where $Z_{\mathrm{ni}}$ denotes the moderating variables (n=1,2); specifically, $Z_{1i}$ is an indicator for whether the farmer has received digital technology training, and $Z_{2i}$ represents the distance from the village to the county administrative center. $\beta_{3}$ and $\beta_{4}$ are the interaction and main effects of the moderators, respectively. All other terms are defined as in Equation 1.

## 4 Results and analysis

### 4.1 Baseline regression analysis

To test the robustness of the model and the correlation among explanatory variables, this study conducts a multicollinearity diagnosis on all control variables. The results show that the variance inflation factors (VIFs) of each variable are significantly lower than 10, and the average VIF value is approximately 1.05, ruling out the possibility of severe collinearity. On this basis, Table 2 presents the regression results of the impact of agricultural insurance on the adjustment of farmers’ planting structure. The specific models are shown in Formulas 1 and 2. Columns (1) to (4) successively add control variables such as household head, household, and village characteristics. Column (5) reports the average marginal effect calculated based on the regression results in Column (4). The variances of the disturbance terms across all columns are significant at the 1% significance level, indicating that there is non-negligible volatility in the latent dependent variables, which further confirms the rationality of the adopted settings. Meanwhile, the agricultural insurance variable is positively significant at the 1% level across all regressions. Specifically, agricultural insurance increases the proportion of staple crops by an average of 24.4%, demonstrating that its agricultural insurance promotes a shift in farmers’ planting structure toward staple food crops. Specifically, on the one hand, agricultural insurance enhances farmers’ confidence in planting staple food crops by diversifying agricultural risks and stabilizing income expectations; on the other hand, insured farmers may show higher institutional trust and policy responsiveness, so they are more inclined to choose the staple food production path strongly supported by the government when making decisions. Thus, Hypothesis H1 is verified, that is, agricultural insurance facilitates grain-oriented adjustment in cropping structure.

**Table 2:** Baseline regression results: effect of agricultural insurance on cropping structure.

Variable	(1)	(2)	(3)	(4)	(5)
	Cropping structure	Cropping structure	Cropping structure	Cropping structure	Average marginal effects
Agricultural insurance	0.568^***^	0.570^***^	0.583^***^	0.564^***^	0.244^***^
	(0.037)	(0.037)	(0.037)	(0.037)	(0.014)
Gender		0.004	0.004	−0.001	−0.000
		(0.082)	(0.082)	(0.082)	(0.036)
Age		0.005^***^	0.005^***^	0.005^***^	0.002^***^
		(0.002)	(0.002)	(0.002)	(0.001)
Education level		0.016	0.012	0.008	0.004
		(0.021)	(0.021)	(0.021)	(0.009)
Village official		−0.061	−0.059	−0.058	−0.025
		(0.044)	(0.044)	(0.044)	(0.019)
Household size			0.015	0.013	0.006
			(0.011)	(0.011)	(0.005)
Total cultivated area			−0.039^**^	−0.036^*^	−0.016^*^
			(0.020)	(0.020)	(0.009)
Low-income household			−0.064	−0.079	−0.034
			(0.055)	(0.055)	(0.024)
Parcel delivery accessibility				−0.089^***^	−0.039^***^
				(0.021)	(0.009)
Urban suburb status				0.029	0.013
				(0.045)	(0.019)
Village collective assets				−0.048^*^	−0.021^*^
				(0.026)	(0.011)
Constant	0.622^***^	0.333^**^	0.302^**^	0.538^***^	
	(0.023)	(0.136)	(0.145)	(0.159)	
var (e. Y)	0.556^***^	0.555^***^	0.553^***^	0.547^***^	
	(0.030)	(0.030)	(0.030)	(0.029)	
Pseudo *R*^2^	0.055	0.057	0.058	0.062	
*N*	2,367	2,367	2,367	2,367	2,367

Robust standard errors are reported in parentheses. ***p < 0.01, **p < 0.05, *p < 0.1.

Based on the regression results in Column (4) of Table 2, further analysis of the impact of each control variable on the adjustment of farmers’ planting structure mainly leads to the following conclusions: First, farmers’ age is significantly and positively associated with the adjustment toward staple crops, indicating that older farmers are more inclined to plant staple food crops. This may reflect their stronger preference for the stability of agricultural production, or due to their higher risk-aversion awareness, they tend to choose staple food crops with smaller income fluctuations and greater policy support to avoid the uncertainty brought by market price fluctuations. Second, at the village level, the coefficient for “parcel delivery accessibility” is significantly negative, indicating an inverse relationship between transportation convenience and grain-oriented cropping. The improvement of transportation and logistics conditions helps to enhance the external sales capacity of agricultural products and reduce transportation costs, making cash crops or high-value-added crops more accessible to the market. Compared with staple food crops, these crops have higher marginal returns, which encourages farmers to adjust their planting structure to pursue higher market returns. This result shows that the adjustment of the agricultural structure is not only driven by policy factors but also deeply affected by infrastructure and market access capabilities.

### 4.2 Robustness checks

**Figure 1:**
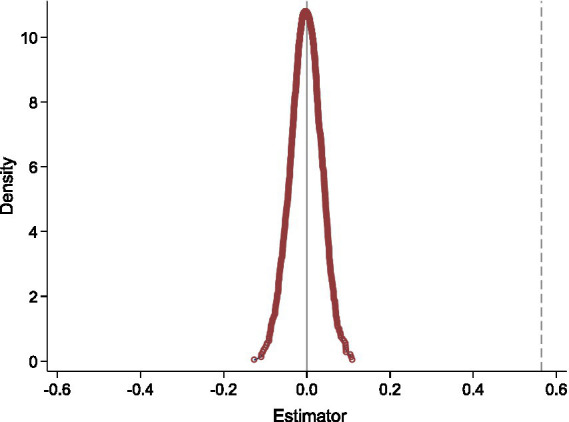
Distribution of simulated coefficients from 1,000 placebo replications.

To further verify the reliability of the estimated effect of agricultural insurance on farmers’ cropping structure adjustment, four robustness checks were conducted: a placebo test, alternative regression model, winsorization of continuous variables, and propensity score matching (PSM). The results are presented in Figure 1 and Table 3.

**Table 3:** Robustness check results: winsorization and PSM matching.

Variable	(1)	(2)	(3)	(4)
	Alternative model	Winsorized sample	Nearest neighbor (1:2)	Kernel matching
Agricultural insurance	1.301^***^	0.565^***^	0.559^***^	0.568^***^
	(0.073)	(0.038)	(0.041)	(0.038)
Control variables	Yes	Yes	Yes	Yes
Constant	0.181	0.544^***^	0.426^**^	0.555^***^
	(0.330)	(0.167)	(0.177)	(0.160)
var (e. Y)		0.548^***^	0.490^***^	0.549^***^
		(0.029)	(0.029)	(0.030)
Pseudo *R*^2^		0.061	0.074	0.063
ATT			Diff = 0.262^***^, T = 14.09	Diff = 0.256^***^, T = 17.70
*N*	2,367	2,367	1824	2,360

Robust standard errors are reported in parentheses. ***p < 0.01, **p < 0.05, *p < 0.1.

#### 4.2.1 Placebo test

To address potential sample selection bias arising from farmers’ self-selection into insurance, a placebo test was conducted following the related approach (31). Specifically, the sample of farmers was randomly shuffled, and pseudo-groups were constructed to generate a counterfactual distribution of estimated coefficients. As shown in Figure 1, the dashed line represents the actual estimated coefficient for agricultural insurance (0.564), while the solid curve shows the distribution of coefficients from 1,000 randomized simulations. The placebo distribution is centered around zero and follows a normal shape, as expected. Importantly, the real coefficient is located far to the right of the simulated values, indicating that the observed effect is unlikely to be due to random chance and further supporting Hypothesis H1.

#### 4.2.2 Alternative regression model

To further verify the robustness of the regression results, this study introduces the fractional logit model as an alternative estimation method, in addition to the baseline two-limit Tobit model. The fractional logit model is suitable for continuous proportional dependent variables ranging between 0 and 1 and allows for direct modeling of the conditional expectation without imposing truncation. Although this model may face certain limitations when handling a large number of boundary values (such as 0 or 1), it remains a widely accepted approach for analyzing fractional data and serves as a useful complement to the baseline specification. As shown in Column (1) of Table 3, the coefficient of the agricultural insurance variable remains positive and statistically significant at the 1% level. This result suggests that the positive effect of agricultural insurance on the proportion of staple crop cultivation remains robust under the alternative model specification.

#### 4.2.3 Winsorization of continuous variables

To mitigate the influence of outliers, we applied a 5% top and bottom winsorization to all continuous variables, including cropping structure, age, total cultivated area, and village collective assets. The regression result is reported in Column (2) of Table 3, showing that the coefficient for agricultural insurance remains at 0.565 and is still statistically significant at the 1% level. This confirms that the main conclusion is not driven by a few extreme observations and is thus robust.

#### 4.2.4 Propensity score matching

To further address sample selection bias, we employed PSM using both nearest-neighbor (1:2) and kernel matching approaches, with all control variables from the baseline regression included as covariates. The results are shown in Columns (3) and (4) of Table 3. The average treatment effect on the treated (ATT) is 0.262 for nearest-neighbor matching and 0.256 for kernel matching, both statistically significant at the 1% level. Additional regression using the matched samples shows that agricultural insurance continues to exert a significant positive effect on cropping structure. These results provide causal support for the effectiveness of agricultural insurance in encouraging farmers to increase the share of staple crops.

### 4.3 Endogeneity test

There may be endogeneity concerns in the relationship between agricultural insurance and farmers’ cropping structure adjustment, primarily arising from two sources. First is the issue of reverse causality: Farmers with a higher proportion of staple crop cultivation are more likely to align with policy priorities and thus more likely to receive insurance coverage and subsidies, increasing their probability of participation. Second is the problem of omitted variable bias: Unobserved individual characteristics such as farmers’ risk preferences and information access may simultaneously influence both their insurance participation and crop choices. To address these endogeneity concerns, this study employs an instrumental variable approach, using “whether the village experienced a natural disaster in the past 3 years” as the instrument. This variable captures the exogenous shock to insurance enrollment behavior, allowing for the identification of a quasi-causal effect of insurance participation. On the one hand, in terms of relevance, natural disasters represent a major source of agricultural risk that significantly raises farmers’ awareness of risk protection, thereby strengthening their motivation to purchase insurance. Farmers suffering losses or facing future production uncertainty after a disaster are more likely to adopt insurance as a coping strategy. A substantial body of literature confirms that disaster experience is a key determinant of insurance uptake. On the other hand, with respect to exogeneity, natural disasters are exogenous shocks driven by environmental conditions and are largely random in their timing and location. The disaster variable used in this study—village-level disaster incidence over the past 3 years—is lagged in time and determined by natural geography rather than by any individual farmer’s crop choices. Thus, the instrument is unlikely to directly affect the cropping structure, satisfying the exclusion restriction by influencing crop choice only through its effect on insurance behavior. In summary, this instrument satisfies both the relevance and exogeneity conditions, making it suitable for identifying the causal impact of agricultural insurance.

Given that the potentially endogenous regressor (agricultural insurance) is binary, we adopt the control function approach. In the first stage, a probit model is used to estimate the probability of insurance participation, and the residual from this regression is constructed as a control function. This residual is then included in the second-stage Tobit model to correct for endogeneity bias. The results are presented in Table 4. Column (1) shows that natural disaster experience has a significant positive effect on insurance participation, supporting instrument relevance. Although the Pseudo R^2^ is 0.0374, the overall model is highly significant (Wald chi^2^ = 96.24, *p* < 0.001), indicating good explanatory power. In supplementary linear probability models, the F-statistic exceeds 10, further ruling out the presence of weak instruments. Column (2) reports the second-stage Tobit regression. Agricultural insurance remains significantly and positively associated with grain-oriented cropping structure, confirming its role as an effective policy tool. Moreover, the residual term is significant at the 1% level, indicating that endogeneity is indeed present and that the control function approach has successfully corrected for it. Overall, the results further strengthen the causal interpretation of agricultural insurance’s role in optimizing cropping structure. These findings affirm that the policy functions not only through external protection but also via internal behavioral incentives.

**Table 4:** Endogeneity test results using control function approach.

Variable	(1)	(2)
	Agricultural insurance (Probit)	Cropping structure (Tobit)
Agricultural insurance		1.399^***^
		(0.091)
Natural disaster experience	0.098^*^	
	(0.055)	
Control variables	Yes	Yes
Residual		−0.914^***^
		(0.087)
Control variables	Yes	Yes
Constant	0.038	0.189
	(0.250)	(0.158)
var (e. Y)		0.513^***^
		(0.027)
Pseudo *R*^2^	0.037	0.086
*N*	2,367	2,367

Robust standard errors are reported in parentheses. ***p < 0.01, **p < 0.05, *p < 0.1.

### 4.4 Moderating effect analysis

Previous results have shown that agricultural insurance significantly promotes grain-oriented adjustment in farmers’ cropping structures. To further identify whether this policy effect varies due to differences in farmers’ capabilities or the external institutional environment, we introduce moderating variables and constructs interaction terms to analyze their impact on the mechanism of agricultural insurance. Specifically, two variables, namely, digital technology training and distance to the county administrative center, are selected to represent farmers’ information capabilities and administrative accessibility, respectively, so as to explore the applicable boundaries and moderating basis of the agricultural insurance policy. The specific model is shown in Formula 3.

**Figure 2:**
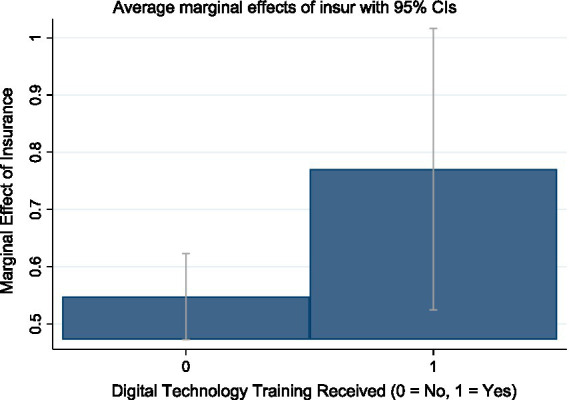
Moderating effect of digital technology training on agricultural insurance.

Column (1) of Table 5 reports the moderating effect of digital technology training. The results show that the coefficient of the interaction term between agricultural insurance and digital technology training is positive and statistically significant at the 10% level, indicating that digital training enhances the impact of agricultural insurance on the shift toward staple crop cultivation. Specifically, when farmers have received computer or mobile-Internet training, the guiding effect of agricultural insurance on their tendency to plant staple grains is more significant, thus verifying Hypothesis H2. Figure 2 further demonstrates the above-mentioned moderating mechanism. It shows that the marginal effect of agricultural insurance is substantially higher among digitally trained farmers. This underscores the importance of information acquisition ability in the incentive pathway of agricultural insurance. Farmers who have received basic digital technology training are generally more likely to obtain agricultural insurance policy content through digital channels, understand the scope of protection and subsidy policies more promptly, and thus make more rational planting choices. In contrast, among farmers with limited information-receiving capabilities, there is a trend of marginal decline in institutional incentives, and it is more difficult to release the policy effect. This finding further confirms that there are differences in the incentive margins of agricultural insurance based on different cognitive capabilities, and the improvement of digital competencies may be a key path to amplify the institutional effect.

**Table 5:** Moderating effect analysis.

Variable	(1)	(2)
	Cropping structure	Cropping structure
Agricultural insurance	0.548^***^	0.354^***^
	(0.038)	(0.061)
Digital technology training	−0.191^*^	
	(0.101)	
Agricultural insurance × digital technology training	0.223^*^	
	(0.128)	
Distance to county government		−0.007^***^
		(0.001)
Agricultural insurance × Distance to county government		0.008^***^
		(0.002)
Control variables	Yes	Yes
Constant	0.561^***^	0.786^***^
	(0.159)	(0.163)
var (e. Y)	0.545^***^	0.535^***^
	(0.029)	(0.029)
Pseudo *R*^2^	0.064	0.071
*N*	2,367	2,367

Robust standard errors in parentheses. ***p < 0.01, **p < 0.05, *p < 0.1.

**Figure 3:**
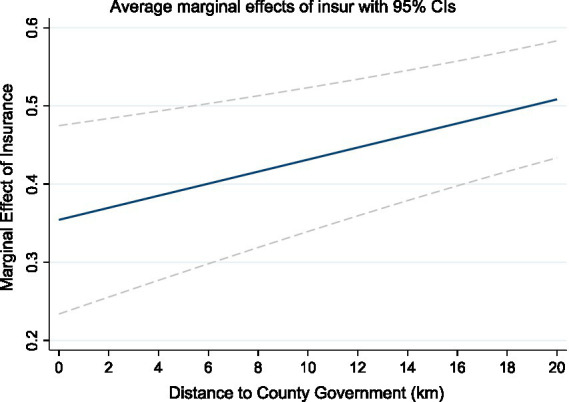
Moderating effect of distance to county government on agricultural insurance.

Column (2) of Table 5 further analyzes the moderating effect of distance to the county government. The interaction term between agricultural insurance and distance to the county government is significantly positive at the 1% level, indicating that the farther the village committee is from the county government, the stronger the promoting effect of agricultural insurance in promoting staple crop cultivation—thereby supporting Hypothesis H3. Figure 3 further shows the trend of the marginal effect of agricultural insurance changing with increasing distance to the county government. It can be seen that as the distance from the county government increases, the marginal effect of agricultural insurance gradually strengthens. This result reveals the important substitution function of agricultural insurance in areas with weak administrative resource coverage. In villages far from the county government, the transmission of policy information is lagged, the supply of technical services is insufficient, and farmers have limited access to other government support policies. In this institutional environment, as a market-oriented mechanism, the risk-protection function of agricultural insurance is more likely to be manifested and has a stronger incentive effect on farmers’ production behaviors. Therefore, in such areas, agricultural insurance not only plays the traditional role of risk buffering but also partially fills the gap in policy supply in the absence of administrative support, having the “institutional substitution” function. This result shows that improving the agricultural insurance system can achieve higher marginal returns in areas with weak institutional coverage, providing important support for regional balanced development.

### 4.5 Heterogeneity analysis

Although the preceding results suggest that agricultural insurance generally promotes the adjustment of farmers’ cropping structures toward staple crops, there exists considerable variation in farmers’ individual capacities, operational conditions, and regional environments. These differences may lead to uneven policy effects across individuals or regions. To address this, we conduct a comprehensive heterogeneity analysis from five perspectives: (1) Education level of the household head, serving as a proxy for cognitive ability and access to information; (2) Total cultivated area, representing farm size and the household’s capacity to withstand risk; (3) Pilot region status for full-cost or income insurance, reflecting variations in insurance coverage levels; (4) Village terrain type, capturing the influence of natural conditions on agricultural decision-making; (5) Geographical region, reflecting differences in policy implementation under varying socioeconomic development levels.

#### 4.5.1 Heterogeneity by education level

Education is a critical factor influencing farmers’ cognitive ability, access to information, and responsiveness to policy interventions (32). To identify the heterogeneous effects of agricultural insurance by education, this study divides household heads into two groups: junior high school and below, and senior high school and above, and conducts separate regressions. The results, presented in Table 6, show that agricultural insurance more effectively promotes the shift toward staple crop cultivation among the more highly educated group.

**Table 6:** Heterogeneous effects of agricultural insurance by education level.

Variable	(1)	(2)
	Junior high school or below	High school or above
Agricultural insurance	0.527^***^	0.817^***^
	(0.039)	(0.104)
Control variables	Yes	Yes
Constant	0.564^***^	0.670
	(0.152)	(0.411)
var (e. Y)	0.547^***^	0.507^***^
	(0.031)	(0.075)
Pseudo *R*^2^	0.056	0.069
*N*	2056	311

Robust standard errors are reported in parentheses. ***p < 0.01, **p < 0.05, *p < 0.1.

This may be attributed to differences in marginal incentives across groups with varying levels of cognitive ability. Farmers with higher education levels tend to have stronger risk perception and a better understanding of policies, allowing them to grasp more accurately the risk compensation function of agricultural insurance in staple crop production. They are more likely to recognize that, under policy support and insurance coverage, staple crop cultivation offers greater income stability and security. As a result, they are more inclined to optimize their cropping structure and shift back to staple crops under manageable risk. In contrast, farmers with lower education levels often face disadvantages in accessing information, interpreting policies, and assessing risks. They may have difficulty fully understanding how agricultural insurance works, perceive weaker policy incentives, and thus rely more on traditional experience and short-term stability in their crop choices.

#### 4.5.2 Heterogeneity by farm size

The management scale of farmers determines their resource allocation ability, risk exposure, and the flexibility of production behavior (33). To explore the heterogeneity of the effects of agricultural insurance under different management scales, based on the World Bank’s definition of the management scale of small-scale farmers (30 mu) and combined with the basic national condition that the per-household arable land area in China is less than 10 mu, this study divides small-scale farmers according to the standards of 10 mu and 30 mu, respectively (34). As shown in Table 7, regardless of which division standard is adopted, agricultural insurance significantly promotes a shift toward staple crop cultivation among smallholders.

**Table 7:** Heterogeneous effects of agricultural insurance by farm size.

Variable	(1)	(2)	(3)	(4)
	<10 mu	≥10 mu	<30 mu	≥30 mu
Agricultural insurance	0.570^***^	0.529^***^	0.563^***^	0.548^***^
	(0.059)	(0.044)	(0.044)	(0.066)
Control variables	Yes	Yes	Yes	Yes
Constant	0.595^**^	0.585^***^	0.393^**^	1.269^***^
	(0.238)	(0.208)	(0.180)	(0.352)
var (e. Y)	0.718^***^	0.373^***^	0.593^***^	0.363^***^
	(0.055)	(0.028)	(0.036)	(0.040)
Pseudo *R*^2^	0.051	0.103	0.060	0.117
*N*	1,333	1,034	1909	458

Robust standard errors are reported in parentheses. ***p < 0.01, **p < 0.05, *p < 0.1.

From an economic perspective, this finding reflects substantial differences in risk preferences, marginal benefits from insurance, and crop selection logic across farm sizes. Smallholders, due to limited land resources, weaker capital reserves, and lower risk-bearing capacity, are more vulnerable to natural disasters and market fluctuations and thus tend to exhibit strong risk aversion and adopt conservative cropping strategies. Agricultural insurance provides income protection and stabilizes expectations, helping alleviate planting constraints arising from risk concerns, and enhancing both the confidence and capacity of smallholders to adjust their cropping structure—particularly by increasing staple crop acreage. In contrast, large-scale farmers, though better equipped in terms of resources and risk management, are more market-oriented in their production behavior. Their crop choices are primarily driven by profit maximization. If agricultural insurance cannot compensate for the relative profitability disadvantage of staple crops, it may have limited influence on their planting decisions.

#### 4.5.3 Heterogeneity by full-cost and income insurance pilot regions

To improve the coverage level of agricultural insurance and stabilize the income of grain producers, China’s Ministry of Finance launched pilot programs for full-cost insurance and income insurance for the three major staple crops in selected provinces starting in 2018. These pilot initiatives aim to address the gap between existing insurance coverage and actual agricultural production costs by covering the full cost of production or offering direct income protection (35). Based on whether a province is part of the pilot program, this study divides the sample into pilot and non-pilot regions and conducts subgroup regressions. As shown in Table 8, the results indicate that agricultural insurance has a stronger effect in promoting the shift toward staple crops in pilot regions.

**Table 8:** Heterogeneous effects of agricultural insurance by full-cost and income insurance pilot regions.

Variable	(1)	(2)
	Pilot region	Non-pilot region
Agricultural insurance	0.560^***^	0.509^***^
	(0.064)	(0.045)
Control variables	Yes	Yes
Constant	0.362	0.693^***^
	(0.279)	(0.190)
var (e. Y)	0.502^***^	0.541^***^
	(0.048)	(0.035)
Pseudo *R*^2^	0.120	0.048
*N*	786	1,581

Robust standard errors are reported in parentheses. ***p < 0.01, **p < 0.05, *p < 0.1.

This finding suggests that as insurance coverage levels improve, agricultural insurance not only performs its traditional role of risk transfer and income protection but also helps reshape farmers’ risk expectations, thereby indirectly guiding more rational agricultural resource allocation and optimizing cropping structure to enhance food security. Moreover, the strengthened protection mechanisms help mitigate production risks arising from price volatility and natural disasters, further encouraging farmers to grow staple crops. This has important implications for advancing agricultural modernization and safeguarding national food security.

#### 4.5.4 Heterogeneity by terrain type

China’s terrain is highly diverse, with uneven distribution of arable land across regions, and agricultural production is significantly shaped by natural conditions (15). The plains of the eastern and central regions feature flat terrain and convenient farming conditions, making them key areas for agricultural production and staple crop cultivation. In contrast, non-plain areas—such as the southwestern hills, the Loess Plateau, and mountainous regions—are characterized by rugged topography, fragmented farmland, lower levels of mechanization, and frequent natural disasters, all of which pose greater uncertainty and risk to agricultural production. As an important exogenous factor in agriculture, terrain not only influences farmers’ crop choices and input decisions but may also significantly affect the incentive effects of agricultural insurance policies. Accordingly, this study divides the sample into two groups—plain and non-plain regions—based on village-level terrain characteristics and conducts separate regressions. The results, shown in Table 9, indicate that agricultural insurance has a significantly stronger effect in promoting adjustments toward staple crop cultivation in non-plain areas.

**Table 9:** Heterogeneous effects of agricultural insurance by terrain type.

Variable	(1)	(2)
	Plain region	Non-plain region
Agricultural insurance	0.412^***^	0.521^***^
	(0.061)	(0.046)
Control variables	Yes	Yes
Constant	0.636^**^	0.585^***^
	(0.260)	(0.196)
var (e. Y)	0.521^***^	0.500^***^
	(0.048)	(0.034)
Pseudo *R*^2^	0.053	0.053
*N*	981	1,386

Robust standard errors are reported in parentheses. ***p < 0.01, **p < 0.05, *p < 0.1.

This difference likely stems from the systematic impact of terrain on agricultural risk levels and production constraints. Non-plain regions commonly experience fragmented farmland, steep slopes, and variable climates, making agricultural production more sensitive to natural conditions. In the absence of other effective risk management tools, farmers in these areas rely more heavily on the risk-buffering function of agricultural insurance and are thus more likely to respond to policy incentives by shifting toward staple crop cultivation. In addition, farmers in non-plain areas generally possess weaker resource endowments and lower risk-bearing capacity. With the support of insurance coverage and related policy measures, they tend to prefer the relative safety and stability of staple crops, which makes the marginal incentive effect of agricultural insurance more pronounced. In contrast, farmers in plain areas benefit from better agricultural infrastructure and greater access to information, enabling them to utilize a wider range of risk management strategies and exercise more autonomy in production decisions. As a result, the marginal impact of agricultural insurance is relatively limited in these regions.

#### 4.5.5 Heterogeneity by region

China spans a vast territory, with significant regional differences in resource endowments. The eastern region benefits from favorable water–heat conditions and a strong agricultural foundation; the central region serves as a major grain-producing area, while the western region faces constraints such as land scarcity, water shortages, and frequent natural disasters, resulting in relatively lagging agricultural development. Due to variations in resource endowments, market environments, and institutional implementation capacity across regions, the effects of agricultural insurance may exhibit significant heterogeneity (36). To explore this, the study conducts regressions based on the standard division of China into eastern, central, and western regions. As shown in Table 10, agricultural insurance exerts a significantly positive effect on the shift toward staple crop cultivation in all three regions but with a clear gradient: The strongest effect is observed in the eastern region, followed by the central, and weakest in the west.

**Table 10:** Heterogeneous effects of agricultural insurance by region.

Variable	(1)	(2)	(3)
	East	Central	West
Agricultural insurance	0.839^***^	0.621^***^	0.296^***^
	(0.100)	(0.081)	(0.042)
Control variables	Yes	Yes	Yes
Constant	0.580	0.853^**^	0.403^**^
	(0.397)	(0.349)	(0.190)
var (e. Y)	0.928^***^	0.565^***^	0.353^***^
	(0.102)	(0.067)	(0.025)
Pseudo *R*^2^	0.101	0.147	0.038
*N*	750	558	1,059

Robust standard errors are reported in parentheses. ***p < 0.01, **p < 0.05, *p < 0.1.

These regional differences likely reflect imbalances in agricultural development, policy transmission efficiency, and institutional conditions. The eastern region has better production infrastructure, a higher level of market development, and a more complete insurance delivery and claims system, enabling agricultural insurance incentives to be more effectively transmitted to farmers’ cropping decisions. The central region, while being a key grain-producing area, lags somewhat behind the east in terms of policy implementation and insurance service quality, leading to a weaker effect. In the western region, poor infrastructure, fragmented farmland, and more complex cropping systems impose greater constraints on insurance implementation. Moreover, in some areas, natural conditions are not suitable for growing the three major staple crops, making it difficult for farmers to adjust their cropping structure toward staples even with policy incentives. Therefore, the effectiveness of agricultural insurance depends not only on institutional design but also on the natural suitability and policy delivery capacity of different regions.

## 5 Discussion and conclusion

### 5.1 Discussion

This study systematically evaluates the effect of agricultural insurance on grain-oriented adjustments in farmers’ cropping structure and, through moderation mechanisms and multi-dimensional heterogeneity analyses, reveals the pathways and policy impacts of agricultural insurance in guiding production decisions. The findings show that agricultural insurance, as a core agricultural risk management tool, not only serves a foundational role in income protection but also exerts a structural incentive effect by optimizing crop allocation and promoting staple crop production. These results support theoretical expectations that agricultural insurance helps stabilize farmers’ risk perceptions (7) and are consistent with the view that policy-based insurance provides behavioral incentive signals (20). The moderation analysis reveals that the incentive effects of agricultural insurance are significantly influenced by farmers’ informational capacity and the institutional environment. On the one hand, for farmers who received digital training—such as basic computer or mobile Internet skills—the effect of insurance on increasing staple crop share is more pronounced. This finding suggests that enhancing digital competence improves the behavioral responsiveness to institutional incentives. The result is consistent with prior research linking digital literacy with greater policy responsiveness (37) and highlights the intermediating role of digital access in policy transmission. On the other hand, the marginal effect of agricultural insurance increases with greater distance from the county administrative center, indicating its compensatory function in institutionally underserved areas. This finding echoes the “substitutive role” of insurance in areas where public service provision is limited (6) and offers empirical evidence for building a more spatially balanced institutional support system. The heterogeneity analysis shows significant variation in the policy’s incentive effects across individual and spatial dimensions. Farmers with higher education levels exhibit greater responsiveness, confirming that stronger cognitive and information-processing skills amplify institutional effects (32, 38). Smallholders, constrained by limited resources and lower risk tolerance, show greater dependence on agricultural insurance and benefit more from its risk-buffering role, consistent with conclusions that small farmers are more likely to gain from such tools (33, 39). At the institutional level, insurance has a stronger impact in pilot regions for full-cost and income insurance, demonstrating that enhancing protection levels reinforces policy orientation (28). Under natural constraints, farmers in non-plain areas are more sensitive to insurance due to higher risks and limited accessibility, highlighting the spatially substitutive role of insurance in these environments (40). Regionally, the strongest effects are observed in the eastern region, followed by central, and weakest in western China—reflecting the combined influence of institutional capacity, information dissemination, and market conditions on policy outcomes (24).

It should be noted that the change in the planting structure not only has significance at the levels of food security and policy response but also relates to deeper-level nutritional security goals. Staple food crops, especially rice and wheat, have long played an irreplaceable core role in maintaining the basic calorie supply for residents, alleviating the risk of rural hunger, and supporting the national nutritional baseline. Promoting the return of staple food planting helps to improve calorie availability and dietary stability, especially in the face of extreme weather and market fluctuations, which has important strategic value and resilience functions. However, at the same time, over-concentration on staple food crops may also compress the planting space for cash crops or minor cereals rich in micronutrients to a certain extent, thus affecting the diversity of the dietary structure and the comprehensiveness of micronutrient intake. In the process of promoting the staple-food-oriented structural optimization, nutritional sensitivity considerations should be incorporated simultaneously, and supporting measures such as biofortification, nutritional crop guidance, and compound planting should be promoted. In this way, the synergistic effect of nutritional balance on the basis of ensuring calorie supply in agricultural structural adjustment can be enhanced, and the goal can be shifted from “having enough to eat” to “eating well.” Overall, as a structural incentive tool, agricultural insurance has effectively increased the proportion of staple food crop planting and played an important role in stabilizing calorie supply. In future policy practice, the concept of nutrition-sensitive agriculture should be further integrated, and the organic unity of staple-food orientation and nutritional security should be achieved through methods such as biofortification and diversified planting.

Despite the preceding discussions, this study still faces certain limitations in terms of data and methodology. First, the data used are cross-sectional, making it difficult to reveal the dynamic change process of the effects of agricultural insurance policies and limiting the identification of long-term impacts. Second, the insurance variables do not distinguish between specific insurance types, insurance amounts, or compensation situations, which may lead to an underestimation of the impact of institutional design differences on farmers’ behavior. In addition, due to data limitations, behavioral mechanisms such as risk perception and institutional trust are difficult to incorporate into the model, affecting the in-depth identification of the policy transmission process. Finally, the study focuses on the adjustment of staple crop cultivation, and extrapolation to other agricultural structure fields still needs to be done with caution. Future research can be expanded in the following aspects: First, combine longitudinal micro-data to analyze the long-term dynamic effects of agricultural insurance policies. Second, introduce more detailed insurance variables, such as insurance amounts, compensation, and insurance participation density, to improve the accuracy of policy identification. Third, incorporate behavioral mechanism variables such as farmers’ risk perception and policy trust to enrich the explanatory framework of institutional incentives. Fourth, expand the research objects to fields such as cash crops and animal husbandry to deepen the understanding of the structural adjustment function of agricultural insurance.

### 5.2 Conclusion

Based on data from the 2020 China Rural Revitalization Survey (CRRS), this study systematically evaluates the impact of agricultural insurance on farmers’ cropping structure adjustment and arrives at three main conclusions: (1) Agricultural insurance significantly promotes grain-oriented adjustment in farmers’ cropping structure. Robustness and endogeneity tests consistently support this conclusion, suggesting that agricultural insurance not only mitigates risk but also serves as an institutional incentive tool for optimizing agricultural structure. (2) Moderation analysis reveals that digital technology training and administrative distance significantly affect the policy effectiveness of agricultural insurance. These reflect the mechanisms of enhanced incentives under improved informational capacity and in contexts of limited institutional accessibility, respectively. (3) Heterogeneity analysis shows that the policy effect of agricultural insurance is more pronounced among farmers with higher education, smaller farm size, in regions with higher protection levels, in complex terrain, and in the eastern region. This indicates that agricultural insurance demonstrates differentiated policy functions under varying cognitive capacities, resource endowments, and spatial conditions. Overall, agricultural insurance, as a structural policy instrument, has played a positive role in stabilizing the supply of staple crops and enhancing the security of calorie intake.

### 5.3 Policy implications

To better leverage the policy potential of agricultural insurance in guiding farmers’ cropping structure optimization and increasing the share of staple crops, institutional reform should be advanced under the dual goals of risk protection and structural incentive. Based on empirical findings and international experience, we offer the following three policy recommendations:

1. Strengthen staple crop orientation and optimize incentive structures. Raise the coverage level and subsidy intensity for staple crops to stabilize farmers’ expectations of grain production. Drawing on the U. S. Federal Crop Insurance Program, which combines “revenue protection and crop targeting,” China should promote full-cost and income insurance in key production areas to enhance the guidance and effectiveness of agricultural insurance. At the same time, nutrition-sensitive agricultural products such as biofortified crops may be included in the insurance coverage to support the coordinated achievement of food security and nutrition goals.
2. Promote service decentralization and digital empowerment to improve institutional accessibility. Expand digital literacy training for farmers, improve online insurance enrollment and claims platforms, and enhance the efficiency of policy delivery. In remote areas with weak service capacity, financial support should be increased, and service coverage expanded. Lessons from India’s “geo-zoned subsidy mechanism” and Kenya’s “mobile microinsurance” provide valuable examples for increasing reach and resilience.
3. Implement differentiated institutional supply to enhance adaptability. For groups with stronger cognitive ability, information services should be strengthened to improve policy responsiveness; for those with weaker resource endowments, subsidy mechanisms should be optimized and participation procedures simplified to lower entry barriers. In areas with relatively high insurance coverage, the depth and stability of protection should be further enhanced; in topographically complex regions, risk assessment systems and location-specific products should be improved; and in the more developed eastern region, institutional innovation and cross-mechanism coordination should be promoted. Drawing on Mexico’s “risk-sharing and zone-based pricing” model can help implement region-specific, targeted policy design.

## Data Availability

The original contributions presented in the study are included in the article/supplementary material, further inquiries can be directed to the corresponding author.
